# The Influence of Intraocular Lens Implantation and Alterations in Blue Light Transmittance Level on the Brain Functional Network Architecture Reorganization in Cataract Patients

**DOI:** 10.3390/brainsci11111400

**Published:** 2021-10-24

**Authors:** Anna Maria Sobczak, Bartosz Bohaterewicz, Magdalena Fafrowicz, Aleksandra Domagalik, Ewa Beldzik, Halszka Oginska, Natalia Golonka, Marek Rekas, Dominik Bronicki, Bożena Romanowska-Dixon, Joanna Bolsega-Pacud, Waldemar Karwowski, Farzad V. Farahani, Tadeusz Marek

**Affiliations:** 1 Department of Cognitive Neuroscience and Neuroergonomics, Institute of Applied Psychology, Jagiellonian University, 30-348 Kraków, Poland; vonfrovitz@gmail.com (M.F.); ewa.beldzik@gmail.com (E.B.); halszka.oginska@gmail.com (H.O.); n.a.golonka@gmail.com (N.G.); tademarek@gmail.com (T.M.); 2 Malopolska Centre of Biotechnology, Jagiellonian University, 30-387 Kraków, Poland; aleksandra.domagalik@uj.edu.pl; 3 Department of Psychology of Individual Differences, Psychological Diagnosis, and Psychometrics, Institute of Psychology, University of Social Sciences and Humanities, 03-815 Warsaw, Poland; 4 Ophthalmology Department, Military Institute of Medicine, 04-349 Warsaw, Poland; mrekas@wim.mil.pl (M.R.); d.bronicki@gmail.com (D.B.); 5 Department of Ophthalmology and Ocular Oncology, Medical College, Jagiellonian University, 31-008 Kraków, Poland; bozena.romanowska-dixon@uj.edu.pl (B.R.-D.); joanna.bolsega@gmail.com (J.B.-P.); 6 Computational Neuroergonomics Laboratory, Department of Industrial Engineering & Management Systems, University of Central Florida, Orlando, FL 32816, USA; wkar@ucf.edu (W.K.); farzad.vasheghani@knights.ucf.edu (F.V.F.); 7 Biostatistics Department, John Hopkins University, Baltimore, MD 21218, USA

**Keywords:** neuroimaging, fMRI, graphs, cataract, blue light

## Abstract

Background: Cataract is one of the most common age-related vision deteriorations, leading to opacification of the lens and therefore visual impairment as well as blindness. Both cataract extraction and the implantation of blue light filtering lens are believed to improve not only vision but also overall functioning. Methods: Thirty-four cataract patients were subject to resting-state functional magnetic resonance imaging before and after cataract extraction and intraocular lens implantation (IOL). Global and local graph metrics were calculated in order to investigate the reorganization of functional network architecture associated with alterations in blue light transmittance. Psychomotor vigilance task (PVT) was conducted. Results: Graph theory-based analysis revealed decreased eigenvector centrality after the cataract extraction and IOL replacement in inferior occipital gyrus, superior parietal gyrus and many cerebellum regions as well as increased clustering coefficient in superior and inferior parietal gyrus, middle temporal gyrus and various cerebellum regions. PVT results revealed significant change between experimental sessions as patients responded faster after IOL replacement. Moreover, a few regions were correlated with the difference in blue light transmittance and the time reaction in PVT. Conclusion: Current study revealed substantial functional network architecture reorganization associated with cataract extraction and alteration in blue light transmittance.

## 1. Introduction

Ageing entails numerous changes affecting the brain and eyes, which are strongly connected with each other functionally and anatomically [1,2]. Cataract is one of the most common age-related vision deteriorations, leading to opacification of the lens and therefore visual impairment and blindness. According to Pascolini and Mariotti’s [3] report for the World Health Organization, cataract is the second (33%) cause of visual impairment and the first cause of blindness (51%). Age-related cataract constitutes the largest group of cataract patients and according to some studies, it may be a manifestation of an extensive degenerative process containing extended brain damage and the subsequent disturbed behavior [4,5]. Due to the accumulation of chromophores the natural lens become more yellow during ageing [6] and, therefore, filter the blue light reaching the retina. This particular part of the spectrum is crucial for entrainment of the circadian system and has significant effects on alerting and cognitive responses [7]. Cataract extraction (cataract surgery) is a surgical procedure involving the removal of a natural lens and implanting the intraocular one (IOL) with a blue light filter or without it (crystal lens). According to the review by Davidson et al. [8], patients with blue light-filtering IOLs “should experience the benefit of overall better quality of vision, reduced glare disability at least in some conditions, and better protection against retinal phototoxicity”. However, some previous studies have shown that in a healthy ageing brain the effect of blue light diminishes, particularly in the areas responsible for vision, alertness regulation and higher executive processes [9]. Nevertheless, previous studies have reported positive impact of cataract surgery [10,11]. For example, Lin et al. [11] observed increased grey matter volume of the visual, cognitive-related, and somatosensory brain areas. The above observations may suggest such improvement in brain functioning as vision-related quality of life, cognitive impairment and depressive state, which are believed to be strongly connected with each other [10]. Normalizing vision after cataract surgery was also suggested to have impact on memory and learning [12]. Apart from that, cataract extraction may also improve cognitive function, but it is still unclear whether it is a matter of improved visual function or cataract surgery as such [13,14]. Essentially, cataract extraction benefits are believed to be extended beyond the visual acuity, as it provides improvements in cognition, emotions and general well-being [15].

Vision impairments affect the overall nervous system functioning, which can be investigated with the use of resting-state fMRI. Graph-theory based analysis is one of the well-known methods to evaluate the global and local functional reorganization of the neuronal network. One of the biggest advantages of conducting graph analysis on fMRI data is the ability to investigate intermediate and high levels of organization across the network as a whole [16]. Thus far, graph theory has been used to quantify abnormality of structural and functional networks in various disorders such as borderline personality disorder [17], autism spectrum disorder [18], social anxiety [19] or even illnesses like cancer [20] and diabetes [21].

The current study is a follow up to our previous work investigating hemodynamic bases of daytime sleepiness, experiencing pleasure as well as positive and negative affect in cataract patients [22]. The purpose of our research is to study the consequences of cataract extraction and change in the amount of blue light that reaches the retina after this intervention on the brain functional network architecture reorganization. We hypothesize that brain activity of the aforementioned participants will vary on a global and local level before and after the implantation of the lens and that it will be associated with the change in blue light transmittance. We assume overall better organization of the functional neural networks after the cataract extraction, IOL implantation and alterations in blue light transmittance. Above results would mean higher integration, synchronization, robustness as well as effectiveness of the functional networks after IOL implantation. We strongly believe the aforementioned analyses can enrich the current knowledge regarding the mechanisms of cognitive deficits associated with vision impairments.

## 2. Materials and Methods

### 2.1. Participants

Ophthalmologists recruited 38 healthy patients with diagnosed cataract, however four of them had to drop out after the first session due to health issues. As a result, a total of 34 participants were subject to fMRI examination before and after cataract extraction and the implantation of an intraocular lens. The mean age of participants was M = 62.3 years old, SD = 9.1 (range 34–74 years old), and the sample consisted of 22 women and 12 men. The participants were subject to fMRI examination 2 weeks before the surgery and 6–12 months after (M = 9.22; SD = 2.66), due to individual recovery process as well as the fact that the patients were localized in the different parts of the country. The patients were recruited by qualified ophthalmologists after being diagnosed with the cataract in the Polish national healthcare system. The inclusion criteria were being diagnosed with a cataract as well as the qualification for the surgery. The exclusion criteria were psychiatric and neurological disorders, lesions and contraindications for magnetic resonance imaging. The study was approved by the Bioethics Commission at the Polish Military Institute of Aviation Medicine, Warsaw, Poland (report number 02/2013; 26 February 2013) and Institute of Applied Psychology at the Jagiellonian University, Cracow, Poland (21 February 2017). It was also conducted in accordance with ethical standards described in the Declaration of Helsinki. Moreover, all participants were informed about the procedure and provided their written consent.

### 2.2. IOL and Blue Light Transmittance

Before IOL implantation, patients had their lens measured yellowing either with the use of fluorophotometry (Ocumetrics Fluorotron Master) or with the Lens Opacities Classification System III. (LOCS III). Transmittance data were obtained directly from the first method and were calculated based on Siik et al. [23] for the LOCS III measurements. There were no data for four patients, thus transmittance level was estimated based on the existing data and age. Ophthalmologists recruited patients who had surgery under the national health care. The IOL type was also chosen by ophthalmologists. Patients had two types of IOL implanted: with blue light filter transmitting 68% of light around 475 nm (Alcon AcrySof^®^ IQ model SN60WF) or so-called crystal lenses transmitting 95% of blue light (HOYA Ltd. model iSert 250, Abbott Medical Optics Inc. model Symphony, Alcon AcrySof^®^ IQ model AU00T0, Akreos^®^ model Adapt AO). The difference between blue light transmittance before and after IOL implantation was calculated for each patient. The eyes with higher transmittance before and after the intervention were taken for this calculation (see Supplementary Materials for raw data).

### 2.3. Behavioral Measurements

At each session, before fMRI scanning participants performed a psychomotor vigilance task (PVT), a widely used test of sustained attention measuring the speed with which subjects respond to a visual stimulus [24,25]. Task was performed on a computer with a 19-inch LCD screen and responses were made with arrow or space keys on the keyboard. Participants were instructed to press a button with index finger as soon as the stimulus appears, which stops the stimulus counter and displays the reaction time (RT) in milliseconds for a 1s period. It was emphasized not to press the button in the absence of stimuli, (in such case a false start warning appeared on the screen). If a reaction was slower than 1 s, the warning “too slow” was presented. The inter-trial interval varied randomly from 2 to 10 s, and the task duration was 5 min, comprising about 42 stimuli. For correct responses mean RT, mean RT for 10% of the fastest responses and mean RT for 10% of the slowest responses were calculated as the most frequently reported PVT outcome metrics [24]. The first three stimuli were discarded from the analysis in each PVT trial. A *t*-test was performed to compare the outcomes between the session before and after the IOL replacement. In order to check whether the difference in performance between sessions is related to change in the blue light transmittance, the correlation analysis was conducted.

### 2.4. MRI Data Acquisition

MRI data were acquired using 3T Siemens Skyra MR System (Siemens Medical Solutions, Erlangen, Germany). Structural images were obtained using sagittal 3D T1-weighted MPRAGE sequence. Total of 10 min functional resting state (rs-fmri) EPI images were acquired using gradient-echo single-shot echo planar imaging sequence with the following parameters: TR = 2000 ms; TE = 27 ms; slice thickness = 3 mm, voxel size = 3 mm^3^, with no gap using 20-channel coil. Total of 37 interleaved transverse slices and 300 volumes were acquired. During the acquisition, participants were instructed to keep their eyes open and to not think about anything in particular.

### 2.5. Imaging Data Preprocessing

The rs-fMRI data processing was performed using Data Processing & Analysis for Brain Imaging (DPABI) V4.3 [26] and SPM 12 (Wellcome Trust Centre for Neuroimaging, UCL, London, UK) both working under MATLAB version R2018a (The MathWorks, Inc., Natick, MA, USA). First 10 time points were discarded due to signal equilibration and then slice timing was conducted. Moreover, realignment with assessment of the voxel specific head motion was conducted. None of the participants displayed movements above 3 mm or 3° in one or more of the orthogonal directions and therefore all patients qualified for further analysis. Then, using standard EPI template functional images were linearly normalized in DARTEL to Montreal Neurological Institute (MNI) space and spatially resampled to 3 × 3 × 3 mm voxel size. The 24 motion parameters derived from the realignment step, white matter as well as cerebrospinal fluid signals and five principal components were removed using principal components analysis integrated in a Component Based Noise Correction Method [27]. The global signal was included due to its potential to provide additional valuable information [28]. The signal was then band-pass filtered (0.01–0.08 Hz) to reduce high-frequency noise and low-frequency drift, such as the respiratory and cardiac rhythms. Finally, the functional data were spatially smoothed with 4mm Full Width at Half Maximum (FWHM) kernel.

### 2.6. Parcellation

The preprocessed data were parcellated using Automated Anatomical Labeling (AAL) atlas which separates the brain into 116 regions [29]. In order to investigate possible between-session differences among Default Mode Network, Salience Network, Basal ganglia Network as well as Higher Visual Network, Primary Visual Network and Visuospatial Network, the authors used templates from FIND lab (http://findlab.stanford.edu/functional_ROIs.html, accessed on 15 November 2020).

### 2.7. Graph Metrics

In order to examine the topological properties of functional brain network for each participant at both global and local levels Graphvar 2.02b and MATLAB version R2018a (The MathWorks, Inc., Natick, MA, USA) were used. Global measures aimed at describing macroscale organization and integration of all nodes in the brain network and included: mean clustering coefficient and assortativity. Local properties were calculated for each individual node (region) separately, reflecting the nodal centrality in the network. In this study, we calculated common local properties such as clustering coefficient and eigenvector centrality (the measures are discussed in detail in https://sites.google.com/site/bctnet/measures/list, accessed on 15 November 2020). Data used for graph measures were not smoothed during preprocessing steps. For each subject, 116 regions of interest (ROIs) were defined according to the AAL atlas [29]. In order to obtain a 116 × 116 undirected binary correlation matrix, mean time course for each region was extracted and then the Pearson coefficients between each pair of ROIs were calculated. In order to exclude the spurious links in interregional connectivity matrices [30], we adopted a thresholding procedure based on the strongest connections, removing the weaker ones [31]. This procedure enables to compare network topology within as well as between participants [32]. Network edges were defined using a sparsity thresholding procedure ranging from 0.1 to 0.5 in steps of 0.05.

### 2.8. Statistical Analysis

Paired *t*-test was used in order to compare graph indexes before and after implantation of an intraocular blue light filter lens. All the results were calculated with 5000 iterations and corrected with the Benjamini and Hochberg [33] False Discovery Rate correction at *p* < 0.05. Paired *t*-test with 5000 iterations as well as non-parametric FDR corrected *p*-value < 0.05 was conducted using Graphvar 2.02b and MATLAB version R2018a (The MathWorks, Inc., Natick, MA, USA). Pearson correlation was calculated in order to investigate the association between graph results and both blue light transmittance and PVT results.

## 3. Results

**Figure 1:**
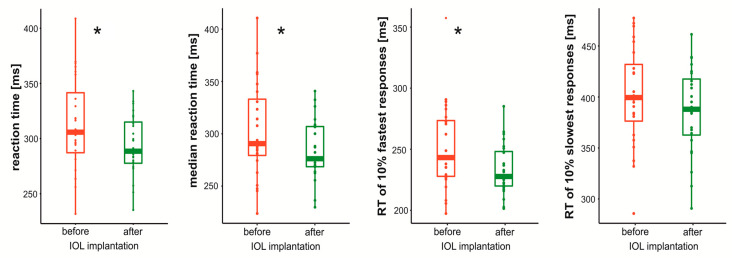
Boxplot showing reaction time distribution before and after Intraocular lens implantation. * Indicate significant difference between sessions.

All PVT outcomes showed significant change between experimental sessions, i.e., patients responded faster after IOL replacement: mean RT (*p* = 0.001, t = 3.65), median RT (*p* = 0.002, t = 3.44) and RT of 10% fastest (*p* = 0.004, t = 3.25) but not the slowest (*p* = 0.072, t = 1.88) responses. Results are presented on Figure 1. The correlation analysis between difference in PVT outcomes between sessions and the difference in blue light transmittance did not reveal significant results (*p* > 0.3).

In case of resting-state fMRI analyses, paired *t*-test revealed significant, FDR corrected (*p* < 0.05) differences in eigenvector centrality values. Among others, the patients manifested higher eigenvector centrality of Vermis 8 before cataract extraction and intraocular lens implantation. Moreover, nodes such as bilateral inferior occipital, bilateral superior parietal, right supramarginal as well as various cerebellum regions turned out to be significantly more important for the whole network before the intraocular lens implantation. Moreover, bilateral supplementary motor area presented a significantly lower tendency for clustering in networks after the surgery, while right superior parietal, left inferior parietal, bilateral middle temporal pole and cerebellum regions clustered to a greater extent. All the results from graph analyses are presented in Table 1.

**Figure 2:**
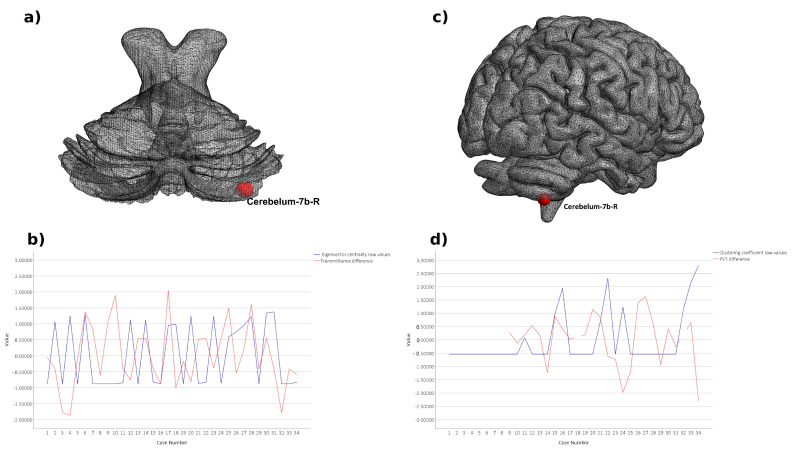
(**a**) Right Cerebelum_7b (aal) ROI rendered on cerebellum surface; (**b**) line plot with Eigenvector centrality raw values and absolute difference in transmittance of each participant; (**c**) Right Cerebelum_7b (aal) ROI rendered on the cortical surface; (**d**) line plot with Clustering coefficient raw values and the difference in PVT of each participant; values for each index were z-score for the purpose of visualization.

**Figure 3:**
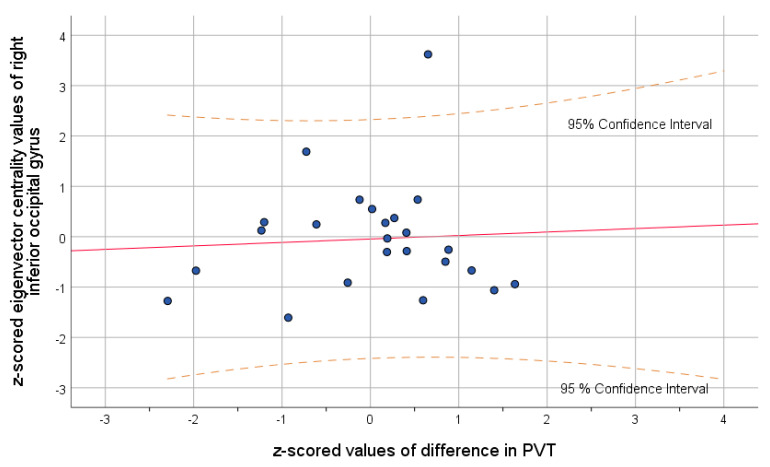
Scatter plot showing the relationship between difference in the reaction time and eigenvector centrality in right inferior occipital gyrus.

The correlation analysis of all significant graph results with the difference in blue light transmittance revealed significant positive correlation on two thresholds between eigenvector centrality in right cerebellum 7b and the difference in the level of the exposure to the blue light (threshold = 0.1, r = 0.37, *p* = 0.032; threshold = 0.3, r = 0.35, *p*_uncorrected_ = 0.043). Correlation analysis of significant graph results with the PVT results revealed significant negative correlation between the difference in the reaction time and clustering coefficient in the right cerebellum 7b in threshold 0.35 (r = −0.34; *p*_uncorrected_ = 0.04), the same structure which was positively correlated with the difference in blue light transmittance. Aforementioned correlations with cerebellum 7b are presented in Figure 2. Moreover, difference in PVT results were negatively correlated with the eigenvector centrality in the right inferior occipital gyrus in two thresholds: 0.3 (r = −0.4; *p*_uncorrected_ = 0.017) and 0.4 (r = −0.36; *p*_uncorrected_ = 0.03). The strongest correlation of altered eigenvector centrality and difference in PVT is visualized in Figure 3.

## 4. Discussion

The current study sought to investigate global and local functional network architecture reorganization of the brain associated with cataract extraction and the difference in blue light transmittance. The proposed local graph metrics revealed the substantial reorganization of functional network architecture, indicating the increase in the clustering coefficient of superior and inferior parietal, middle temporal as well as various cerebellar regions. Large clustering coefficient is reported to be characteristic for a small-world network [34] and creating small worlds in neuronal networks is thought to be a property of healthy brain as the loss of small-worldness is a well-known signature of Alzheimer’s disease [35] and schizophrenia [36]. Moreover, larger clustering coefficient allows differentiating the healthy participants from the ones with an early onset of neurodegenerative dementia [37]. In addition, Masuda et al. [38] revealed significant decline in the clustering coefficient associated with age. The aforementioned reports are congruent with our results, showing considerable increase in the local clustering coefficient after cataract extraction and intraocular lens implantation primarily among the elderly patients. It indicates higher integration and better functioning of brain networks. Larger clustering coefficient in inferior and superior parietal gyrus is probably associated with functional recovery as well as visual restoration, which was already established in the study of Lin et al. [11]. The authors proved that after the cataract surgery, both visual and cognitive functions can be not only enhanced but even fully reversed to the normal level of functioning. Our other results from the bilateral middle temporal poles also stay in line with the literature as this specific region is believed to play a significant role in conceptual processing of visual objects [39]. However, apparently visual improvement is not the only pronounced effect associated with cataract extraction and intraocular lens implantation. The enlarged clustering coefficient in the cerebellum 7b, 8a, Crus 2 as well as vermis 8 indicates considerable functional reorganization associated with motor and cognition alterations. For instance, cerebellum crus 2 is, among others, related to emotional cognition [40] which is reported to boost after cataract extraction [15]. Vermis 8, in turn, is responsible for bodily posture and locomotion, considered to be impaired among people with visual deficits [41,42]. In addition, the current study provides results proving cognitive enhancement caused by cataract surgery. The analysis of local graph metrics revealed larger clustering coefficient in the cerebellum 7b and 8a, which are strongly involved in visual working memory and visual attention tasks. Moreover, the study of Brissenden et al. [43] shows that the above cerebellum areas manifest intrinsic functional connectivity with dorsal attention network. Noteworthy, clustering coefficient in cerebellum 7b turned out to be negatively correlated with the difference in the results of PVT, a widely known test of the sustained attention, which confirms association of cerebellum 7b with the attention network. Concluding, the increased integration of aforementioned regions proves not only visual but also motor and cognitive-related improvement associated with cataract extraction as well as intraocular lens implantation.

Alteration in eigenvector centrality is another proof for significant functional network architecture reorganization. Eigenvector centrality is a self-referential measure of centrality. Nodes with high eigenvector centrality are connected to other nodes with high eigenvector centrality. It means that the node is important for the network but at the same time is connected to other nodes which are very important for the network. The local graph measure allowed identifying significant changes in prominent regions in the hierarchy of brain networks. Eigenvector centrality of the neuron is thought to positively correlate with its relative firing rate [44]. According to the spiking neural network model, increased firing rate constitutes a compensatory mechanism which prevents the disruption of neural network homeostasis after progressive loss of synapses [45,46]. The above results are congruent with the previous study reporting higher eigenvector centrality in participants with longer alcohol dependence [47]. The current study revealed increased eigenvector centrality in bilateral inferior occipital gyrus, bilateral superior parietal gyrus as well as various cerebellum regions in cataract patients before cataract extraction and intraocular lens implantation. The above results may contribute to a spiking neural network model, as both occipital and parietal regions are believed to present impaired functioning in cataract patients before the surgery [11]. Moreover, most of the cerebellum regions with increased centrality before the surgery are the same ones which showed increased clustering coefficient and thereby higher integration after the cataract extraction. The association between altered eigenvector centrality and functional reorganization patterns are still not fully understood and should be further investigated.

In addition, eigenvector centrality in cerebellum 7b turned out to be positively correlated with the difference in the level of blue light transmittance. The above region is thought to be associated with non-motor representations in the brain [48]. The previous study, conducted on 228 healthy subjects revealed strong resting-state functional connectivity between cerebellum 7b and salience network [49], which is reported to be responsible for detecting relevant stimuli as well as coordinating the respective brain response [50]. Moreover, Brissenden et al. [43] reported that the same cerebellum region is functionally connected with the dorsal attention network. The previous results prove cerebellum 7b is related to attention and processing sensory-motor information. Thereby, the current research is congruent with other previous studies pointing to the association between blue light exposure and alertness for external stimuli as well as overall attention [51,52]. Summarizing, blue light transmittance proves to be related to functional reorganization in cerebellum 7b, the region strongly associated with structures which play a significant role in attention and integration of external stimuli. Interestingly, eigenvector centrality in the right inferior occipital gyrus after the IOL implantation turned out to be negatively correlated with the difference in reaction time in psychomotor vigilance test. Above relation means that the lower eigenvector centrality after the cataract extraction, the faster patients reacted to the salient stimuli on the screen after the surgery. Aforementioned results stay in line with the literature considering better visual and attentional performance to be a consequence of cataract extraction [53]. Importantly, improvement of visual acuity is the most reported outcome after the cataract extraction, there are studies showing that the procedure can significantly improve brain functions as a function of increased gray matter volume in brain areas related with cognitive and visual functions [11].

## 5. Limitation

The current study has the restricted sample size and further study should consider extending it. Secondly, the sample size is not equivalent in the case of gender and the age range could be smaller. Moreover, fMRI studies struggle with the low time resolution, however our time repetition had been established to 1000 TR, hence the limitation is minimalized. In addition, both local and global graph metrics have been chosen according to the best authors knowledge, however the process of selection was still arbitrary because there are a lot of other metrics and none of them is believed to be the best index for describing neural reorganization. Finally, future studies should collect more demographical data as well as cognitive measurements.

## 6. Conclusions

Our results reveal tremendous functional network architecture reorganization of neuronal networks caused by cataract extraction, intraocular lens implantation as well as altered blue light transmittance. To the best of our knowledge this is the first report addressing this problem, based on graph theory and conducted with the use of resting state fMRI data.

## Figures and Tables

**Table 1:** List of brain ROIs with clustering coefficient and eigenvector centrality values before and after the intraocular lens implantation.

	*p*-Value			
ROI (Names)	AAL Label	Threshold	Clustering Coefficient	Eigenvector Centrality
**Preoperative-Postoperative**				
Left Supplementary Motor Area	SMA.L	0.2	0.035	
Right Supplementary Motor Area	SMA.R	0.4	0.027	
Left Inferior Occipital Gyrus	IOG.L	0.4		0.034
Right Inferior Occipital Gyrus	IOG.R	0.3		0.009
		0.4		0.02
		0.5		0.015
Left Superior Parietal Gyrus	SPG.L	0.15		0.042
		0.2		0.017
		0.3		0.038
Right Superior Parietal Gyrus	SPG.R	0.15		0.016
Right Supramarginal Gyrus	SMG.R	0.15		0.004
		0.35		0.029
Left Cerebellum Crus 2	CRBLCrus2.L	0.2		0.046
		0.3		0.019
		0.4		0.014
		0.5		0.016
Left Cerebellum 7b	CER7b.L	0.35		0.024
Right Cerebellum 7b	CER7b.R	0.1		0.018
		0.15		0.02
		0.2		0.017
		0.25		0.027
		0.3		0.04
Left Cerebellum 8	CER8.L	0.1		0.03
Right Cerebellum 8	CER8.R	0.15		0.048
		0.45		0.03
Left Cerebellum 9	CER9.L	0.45		0.006
Right Cerebellum 9	CER9.R	0.25		0.011
Vermis 8	VER8	0.1		0.0002
**Postoperative-Preoperative**				
Right Superior Parietal Gyrus	SPG.R	0.45	0.027	
		0.5	0.046	
Left Inferior Parietal Gyrus	IPL.L	0.2	0.022	
		0.25	0.021	
		0.35	0.014	
		0.4	0.014	
Temporal Pole: Left Middle Temporal Gyrus	TPOmid.L	0.4	0.006	
		0.45	0.008	
		0.5	0.014	
Temporal Pole: Right Middle Temporal Gyrus	TPOmid.R	0.35	0.023	
Left Cerebellum Crus 2	CRBLCrus2.L	0.15	0.016	
		0.2	0.015	
		0.25	0.048	
		0.3	0.016	
Right Cerebellum Crus 2	CRBLCrus2.R	0.1	0.022	
		0.25	0.015	
Right Cerebellum 7b	CER7b.R	0.35	0.049	
Left Cerebellum 8	CER8.L	0.15	0.048	
Left Cerebellum 10	CER10.L	0.5	0.01	
Vermis 8	VER8	0.2	0.033	
		0.25	0.034	
		0.3	0.023	
		0.35	0.025	
		0.4	0.022	

## Data Availability

The data can be shared upon the request.

## Supplementary Materials

The following are available online at https://www.mdpi.com/article/10.3390/brainsci11111400/s1, Table S1: Level of blue light transmittance before and after cataract extraction.

## References

[1] Schnitzer M.J., Meister M. (2003). Multineuronal Firing Patterns in the Signal from Eye to Brain. Neuron.

[2] Chen C., Bickford M.E., Hirsch J.A. (2016). Untangling the Web between Eye and Brain. Cell.

[3] Pascolini D., Mariotti S.P. (2011). Global estimates of visual impairment: 2010. Br. J. Ophthalmol..

[4] Linn L., Kahn R.L., Coles R., Cohen J., Marshall D., Weinstein E.A. (1953). Patterns of behavior disturbance following cataract extraction. Am. J. Psychiatry.

[5] Lai S.-W., Lin C.-L., Liao K.-F. (2014). Cataract may be a non-memory feature of Alzheimer’s disease in older people. Eur. J. Epidemiol..

[6] Kessel L., Lundeman J.H., Herbst K., Andersen T.V., Larsen M. (2010). Age-related changes in the transmission properties of the human lens and their relevance to circadian entrainment. J. Cataract. Refract. Surg..

[7] Cajochen C. (2007). Alerting effects of light. Sleep Med. Rev..

[8] Davison J.A., Patel A.S., Cunha J.P., Schwiegerling J., Muftuoglu O. (2011). Recent studies provide an updated clinical perspective on blue light-filtering IOLs. Graefe’s Arch. Clin. Exp. Ophthalmol..

[9] Daneault V., Hébert M., Albouy G., Doyon J., Dumont M., Carrier J., Vandewalle G. (2014). Aging Reduces the Stimulating Effect of Blue Light on Cognitive Brain Functions. Sleep.

[10] Ishii K., Kabata T., Oshika T. (2008). The Impact of Cataract Surgery on Cognitive Impairment and Depressive Mental Status in Elderly Patients. Am. J. Ophthalmol..

[11] Lin H., Zhang L., Lin D., Chen W., Zhu Y., Chen C., Chan K.C.W., Liu Y., Chen W. (2018). Visual Restoration after Cataract Surgery Promotes Functional and Structural Brain Recovery. EBioMedicine.

[12] Fagerström R. (1992). Correlations of Memory and Learning with Vision in Aged Patients before and after a Cataract Operation. Psychol. Rep..

[13] Hall T.A., McGwin G., Owsley C. (2005). Effect of cataract surgery on cognitive function in older adults. J. Am. Geriatr. Soc..

[14] Fukuoka H., Sutu C., Afshari N.A. (2016). The impact of cataract surgery on cognitive function in an aging population. Curr. Opin. Ophthalmol..

[15] Gray C.S., Karimova G., Hildreth A.J., Crabtree L., Allen D., O’Connell J.E. (2006). Recovery of visual and functional disability following cataract surgery in older people: Sunderland Cataract Study. J. Cataract. Refract. Surg..

[16] Medaglia J.D. (2017). Functional Neuroimaging in Traumatic Brain Injury: From Nodes to Networks. Front. Neurol..

[17] Xu T., Cullen K.R., Mueller B., Schreiner M.W., Lim K.O., Schulz S.C., Parhi K.K. (2016). Network analysis of functional brain connectivity in borderline personality disorder using resting-state fMRI. NeuroImage Clin..

[18] Sadeghi M., Khosrowabadi R., Bakouie F., Mahdavi H., Eslahchi C., Pouretemad H. (2017). Screening of autism based on task-free fMRI using graph theoretical approach. Psychiatry Res. Neuroimaging.

[19] Xing M., Tadayonnejad R., MacNamara A., Ajilore O., DiGangi J., Phan K.L., Leow A., Klumpp H. (2016). Resting-state theta band connectivity and graph analysis in generalized social anxiety disorder. NeuroImage Clin..

[20] Bromis K., Gkiatis K., Karanasiou I., Matsopoulos G., Karavasilis E., Papathanasiou M., Efstathopoulos E., Kelekis N., Kouloulias V. (2017). Altered Brain Functional Connectivity in Small-Cell Lung Cancer Patients after Chemotherapy Treatment: A Resting-State fMRI Study. Comput. Math. Methods Med..

[21] Macpherson H., Formica M., Harris E., Daly R.M. (2017). Brain functional alterations in Type 2 Diabetes—A systematic review of fMRI studies. Front. Neuroendocr..

[22] Sobczak A.M., Bohaterewicz B., Fafrowicz M., Zyrkowska A., Golonka N., Domagalik A., Beldzik E., Oginska H., Rekas M., Bronicki D. (2021). Brain Functional Network Architecture Reorganization and Alterations of Positive and Negative Affect, Experiencing Pleasure and Daytime Sleepiness in Cataract Patients after Intraocular Lenses Implantation. Brain Sci..

[23] Siik S., Chylack L.T., Friend J., Wolfe J., Teikari J., Nieminen H., Airaksinen P.J. (1999). Lens autofluorescence and light scatter in relation to the lens opacities classification system, LOCS III. Acta Ophthalmol. Scand..

[24] Dinges D.F., Powell J.W. (1985). Microcomputer analyses of performance on a portable, simple visual RT task during sustained operations. Behav. Res. Methods Instrum. Comput..

[25] Basner M., Dinges D.F. (2011). Maximizing Sensitivity of the Psychomotor Vigilance Test (PVT) to Sleep Loss. Sleep.

[26] Yan C.-G., Wang X.-D., Zuo X.-N., Zang Y.-F. (2016). DPABI: Data Processing & Analysis for (Resting-State) Brain Imaging. Neuroinformatics.

[27] Behzadi Y., Restom K., Liau J., Liu T.T. (2007). A component based noise correction method (CompCor) for BOLD and perfusion based fMRI. NeuroImage.

[28] Liu T.T., Nalci A., Falahpour M. (2017). The global signal in fMRI: Nuisance or Information?. NeuroImage.

[29] Mazoyera N., Landeau B., Papathanassiou D., Crivello F., Etard O., Delcroix N., Tzourio-Mazoyer N., Joliot M. (2002). Automated Anatomical Labeling of Activations in SPM Using a Macroscopic Anatomical Parcellation of the MNI MRI Single-Subject Brain. NeuroImage.

[30] Power J.D., Cohen A., Nelson S.M., Wig G.S., Barnes K.A., Church J., Vogel A.C., Laumann T.O., Miezin F.M., Schlaggar B.L. (2011). Functional Network Organization of the Human Brain. Neuron.

[31] Heuvel M.P.V.D., de Lange S.C., Zalesky A., Seguin C., Yeo B.T., Schmidt R. (2017). Proportional thresholding in resting-state fMRI functional connectivity networks and consequences for patient-control connectome studies: Issues and recommendations. NeuroImage.

[32] Gamboa O., Tagliazucchi E., von Wegner F., Jurcoane A., Wahl M., Laufs H., Ziemann U. (2014). Working memory performance of early MS patients correlates inversely with modularity increases in resting state functional connectivity networks. NeuroImage.

[33] Benjamini Y., Hochberg Y. (1995). Controlling the False Discovery Rate: A Practical and Powerful Approach to Multiple Testing. J. R. Stat. Soc. Ser. B (Methodol.).

[34] Watts D.J., Strogatz S.H. (2011). Collective dynamics of ‘small-world’ networks. The Structure and Dynamics of Networks.

[35] Brier M.R., Thomas J.B., Fagan A.M., Hassenstab J., Holtzman D.M., Benzinger T.L., Morris J.C., Ances B.M. (2014). Functional connectivity and graph theory in preclinical Alzheimer’s disease. Neurobiol. Aging.

[36] Liu Y., Liang M., Zhou Y., He Y., Hao Y., Song M., Yu C., Liu H., Liu Z., Jiang T. (2008). Disrupted small-world networks in schizophrenia. Brain.

[37] Filippi M., Basaia S., Canu E., Imperiale F., Meani A., Caso F., Magnani G., Falautano M., Comi G., Falini A. (2017). Brain network connectivity differs in early-onset neurodegenerative dementia. Neurology.

[38] Masuda N., Sakaki M., Ezaki T., Watanabe T. (2018). Clustering Coefficients for Correlation Networks. Front. Aging Neurosci..

[39] Davey J., Cornelissen P.L., Thompson H.E., Sonkusare S., Hallam G.P., Smallwood J., Jefferies E. (2015). Automatic and Controlled Semantic Retrieval: TMS Reveals Distinct Contributions of Posterior Middle Temporal Gyrus and Angular Gyrus. J. Neurosci..

[40] Van Overwalle F., Ma Q., Heleven E. (2020). The posterior crus II cerebellum is specialized for social mentalizing and emotional self-experiences: A meta-analysis. Soc. Cogn. Affect. Neurosci..

[41] Patla A.E. (1997). Understanding the roles of vision in the control of human locomotion. Gait Posture.

[42] Inoue S., Kawashima M., Hiratsuka Y., Nakano T., Tamura H., Ono K., Murakami A., Tsubota K., Yamada M. (2018). Assessment of physical inactivity and locomotor dysfunction in adults with visual impairment. Sci. Rep..

[43] Brissenden J.A., Levin E.J., Osher D.E., Halko M.A., Somers D.C. (2016). Functional Evidence for a Cerebellar Node of the Dorsal Attention Network. J. Neurosci..

[44] Fletcher J.M., Wennekers T. (2018). From Structure to Activity: Using Centrality Measures to Predict Neuronal Activity. Int. J. Neural Syst..

[45] Skouras S., Falcon C., Tucholka A., Rami L., Sanchez-Valle R., Lladó A., Gispert J.D., Molinuevo J.L. (2019). Mechanisms of functional compensation, delineated by eigenvector centrality mapping, across the pathophysiological continuum of Alzheimer’s disease. NeuroImage: Clin..

[46] Bachmann C., Tetzlaff T., Duarte R., Morrison A. (2020). Firing rate homeostasis counteracts changes in stability of recurrent neural networks caused by synapse loss in Alzheimer’s disease. PLoS Comput. Biol..

[47] Sjoerds Z., Stufflebeam S.M., Veltman D.J., Brink W.V.D., Penninx B.W.J.H., Douw L. (2015). Loss of brain graph network efficiency in alcohol dependence. Addict. Biol..

[48] Guell X., Schmahmann J.D., De Gabrieli J., Ghosh S.S. (2018). Functional gradients of the cerebellum. eLife.

[49] Sang L., Qin W., Liu Y., Han W., Zhang Y., Jiang T., Yu C. (2012). Resting-state functional connectivity of the vermal and hemispheric subregions of the cerebellum with both the cerebral cortical networks and subcortical structures. NeuroImage.

[50] Seeley W.W. (2019). The Salience Network: A Neural System for Perceiving and Responding to Homeostatic Demands. J. Neurosci..

[51] Lehrl S., Gerstmeyer K., Jacob J.H., Frieling H., Henkel A.W., Meyrer R., Wiltfang J., Kornhuber J., Bleich S. (2007). Blue light improves cognitive performance. J. Neural Transm..

[52] Chellappa S., Steiner R., Blattner P., Oelhafen P., Götz T., Cajochen C. (2011). Non-Visual Effects of Light on Melatonin, Alertness and Cognitive Performance: Can Blue-Enriched Light Keep Us Alert?. PLoS ONE.

[53] Wan Y., Yang J., Ren X., Yu Z., Zhang R., Li X. (2020). Evaluation of eye movements and visual performance in patients with cataract. Sci. Rep..

