# Supplementary material for: The Influence of Intraocular Lens Implantation and Alterations in Blue Light Transmittance Level on the Brain Functional Network Architecture Reorganization in Cataract Patients
Source: Brain Sci. 2021 Oct 24;11(11):1400. doi: 10.3390/brainsci11111400 (PMC8615544; doi:10.3390/brainsci11111400)
Supplement: Supplementary file 1 [file brainsci-11-01400-s001.zip › brainsci-1328104-supplementary.pdf]

**Supplementary Table S1.** Level of blue light transmittance before and after cataract extraction.

| No | ID | Gender | Age | transmittance before cataract extraction | | transmittance after cataract extraction | | difference in transmittance before and after cataract extraction |
|---|---|---|---|---|---|---|---|---|
| | | | | RIGHT eye | LEFT eye | RIGHT eye | LEFT eye | |
| **1** | sb141 | M | 59 | 56 | 70 | 68 | 68 | -2 |
| **2** | sb140 | K | 67 | 71 | 70 | 95 | 68 | 24 |
| **3** | sb138 | K | 64 | 95 | 75 | 95 | 68 | 0 |
| **4** | sb134 | K | 47 | 83 | 70 | 68 | 95 | 12 |
| **5** | sb132 | M | 72 | 73 | 73 | 68 | 68 | -5 |
| **6** | sb131 | M | 66 | 52 | 65 | 68 | 68 | 3 |
| **7** | sb130 | M | 67 | 62 | 75 | 68 | 68 | -7 |
| **8** | sb129 | K | 61 | 60 | 64 | 95 | 68 | 31 |
| **9** | sb128 | K | 62 | 74 | 72 | 68 | 68 | -6 |
| **10** | sb127 | K | 34 | 93 | 96 | 68 | ** no intervention* | 0 |
| **11** | sb118 | K | 62 | 72 | 72 | 68 | 68 | -5 |
| **12** | sb109 | K | 47 | 79 | 79 | 95 | 95 | 16 |
| **13** | sb107 | M | 62 | 72 | 72 | 95 | 95 | 22 |
| **14** | sb106 | K | 57 | 66 | 66 | 70 | ** no intervention* | 4 |
| **15** | sb105 | K | 60 | 86 | 74 | 68 | 68 | -18 |
| **16** | sb103 | K | 73 | 85 | 85 | 68 | 68 | -17 |
| **17** | sb102 | K | 59 | 92 | 93 | ** no intervention* | 68 | 0 |
| **18** | sb101 | K | 67 | 64 | 33 | 68 | 68 | 4 |
| **19** | sb152 | K | 67 | 70 | 70 | 68 | 68 | -2 |
| **20** | sb142 | M | 38 | 76 | 87 | 95 | 95 | 8 |
| **21** | sb114 | M | 65 | 50 | 45 | 68 | 68 | 18 |
| **22** | sb112 | K | 72 | 71 | 71 | 68 | 68 | -3 |
| **23** | sb144 | M | 68 | 63 | 70 | 95 | 95 | 25 |
| **24** | sb149 | K | 59 | 84 | 85 | 68 | 68 | -17 |
| **25** | sb139 | K | 66 | 83 | 83 | 95 | 95 | 12 |
| **26** | sb137 | K | 61 | 66 | 83 | 95 | 95 | 12 |
| **27** | sb124 | K | 73 | 83 | 66 | 95 | 95 | 12 |
| **28** | sb122 | K | 69 | 66 | 83 | 95 | 95 | 12 |
| **29** | sb145 | M | 74 | 34 | 95 | 95 | 95 | 0 |
| **30** | sb146 | M | 65 | 83 | 83 | 95 | 95 | 12 |
| **31** | sb147 | M | 60 | 66 | 95 | 95 | 95 | 0 |
| **32** | sb150 | M | 63 | 66 | 95 | 95 | 95 | 0 |
| **33** | sb117 | K | 61 | 95 | 95 | 95 | 95 | 0 |
| **34** | sb116 | K | 70 | 66 | 66 | 95 | 95 | 29 |
